# Prostate Cancer Incidence and Mortality: Global Status and Temporal Trends in 89 Countries From 2000 to 2019

**DOI:** 10.3389/fpubh.2022.811044

**Published:** 2022-02-16

**Authors:** Le Wang, Bin Lu, Mengjie He, Youqing Wang, Zongping Wang, Lingbin Du

**Affiliations:** ^1^Department of Cancer Prevention, Cancer Hospital of the University of Chinese Academy of Sciences (Zhejiang Cancer Hospital), Hangzhou, China; ^2^Office of Cancer Screening, National Cancer Center/National Clinical Research Center for Cancer/Cancer Hospital, Chinese Academy of Medical Sciences and Peking Union Medical College, Beijing, China; ^3^Department of Nutrition and Food Safety, Zhejiang Provincial Center for Disease Control and Prevention, Hangzhou, China; ^4^Department of Urology, Cancer Hospital of the University of Chinese Academy of Sciences (Zhejiang Cancer Hospital), Hangzhou, China

**Keywords:** prostate cancer, incidence, mortality, screening, prevention

## Abstract

**Aims:**

To evaluate current status of prostate cancer incidence and mortality worldwide, and compare the global trends of incidence and mortality in the past two decades and in the most recent period.

**Methods:**

Data on the incidence and mortality of prostate cancer for 174 countries in 2020 were obtained from the GLOBOCAN 2020 database, and associations with the human development index (HDI) were evaluated. Data for trend analyses in 89 countries from 2000 to 2019 were retrieved from the Global Burden of Disease 2019 platform. Age standardized incidence rate (ASIR) and mortality rate (ASMR) were calculated by using the Segi's population. The average annual percent changes (AAPC) of ASIRs and ASMRs were evaluated by joinpoint regression analysis.

**Results:**

A total of 1 414 259 new cases of prostate cancer and 375 304 related deaths were reported in 2020 globally. HDI was positively correlated with ASIRs (*P* < 0.001) and negatively correlated with ASMRs (*P* < 0.001). In the past two decades, ASIRs have been increasing in 65 countries, stable in 15 countries and decreasing in 9 countries, and ASMRs have been increasing in 19 countries, stable in 25 countries and decreasing in 45 countries, respectively. In the most recent period, 44 countries have increasing ASIRs, and 32 countries have decreasing ASMRs, respectively. For instance, in the United States of America, the AAPC of ASIRs significantly decreased by 0.62% and ASMRs significantly decreased by 1.22% from 2000 to 2019, while the AAPC from 2015 to 2019 significantly increased by 0.49% for ASIRs and significantly increased by 0.48% for ASMRs.

**Conclusion:**

The magnitude of increasing incidence and decreasing mortality of prostate cancer is attenuated in the recent period. Further study is needed to analyze the absolute effect of risk factors, PSA screening and treatment.

## Background

Prostate cancer is the second most commonly diagnosed cancer and the fifth leading cause of cancer death among men worldwide, with an estimated 1,414,000 new cancer cases and 375,304 deaths in 2020. Prostate cancer is the most frequently diagnosed cancer in 112 countries, and the leading cause of cancer death in 48 countries (1). It is worth noting that the burden of prostate cancer is supposed to increase owing to the population aging and economic growth (2).

Advancing age, black race, and family history are well-established risk factors for prostate cancer (3). Meanwhile, more lifestyle and dietary risk factors that might increase the risk of prostate cancer have been consecutively put forward, like obesity (4), fitness (5), diabetes mellitus (6), dietary patterns (7), and supplementation with vitamin E (8). Moreover, human development index (HDI), a summary measure of average achievement in key dimensions of human development for each country including life expectancy at birth, education index, and gross national income per capita (9), has demonstrated an impact on the incidence and mortality of prostate cancer (1–3).

Prostate-specific antigen (PSA) based screening has been adopted in several developed countries since 1990's, and downward trend of mortality rate from prostate cancer has been demonstrated in quite a few countries like United States of America (USA) (10), Canada (11), United Kingdom (UK) (12), and Japan (13). As current evidences on the benefit and harm of PSA screening from randomized controlled trials have not yet reached agreement (14–17), intense discussions on the pros and cons of PSA screening are still in progress, and corresponding recommendations for or against the PSA screening are changing frequently in different screening guidelines from a few studies (18–24). Changes in recommendations from guidelines posed obvious impact in the screening patterns and burden of prostate cancer, of which the most typical example is the recommendations from the US Preventive Services Task Force (USPSTF). In 2012, the USPSTF recommended against PSA-based screening for prostate cancer for all men regardless of age (25), whereafter several studies found that screening rates decreased while incidence of advanced prostate cancer increased (26–30). Then in 2018, the USPSTF recommended discussion of the potential benefits and harms of screening with their clinician for men aged 55–69 years (31). Subsequently, a few more screening guidelines have been published in support of PSA screening (32–34), whereas the impact on incidence and mortality of prostate cancer has not yet been determined.

Therefore, this study aimed to summarize the most up-to-date global incidence and mortality for prostate cancer based on the GLOBOCAN 2020 database, and analyze temporal trends in incidence and mortality of prostate cancer from 2000 to 2019 by using consecutive data from the Global Burden of Disease (GBD) 2019 platform, which in combination are supposed to be useful for decision-making on the primary and secondary prevention strategy for prostate cancer.

## Methods

### Data Source

Current status of incidence and mortality rates of prostate cancer in 2020 was analyzed by obtaining data of 185 countries or territories from the GLOBOCAN 2020 (35). Prostate cancer was coded C61 by using the International Classification of Disease (ICD, 10th revision, version 2010). Data on the HDI for each country was retrieved from the United Nations Development Program, from which HDIs in 2019 were available for 174 of the 185 countries (9). Finally, those 174 countries were included in the cross-sectional correlation analyses between the burden of prostate cancer and the HDI. According to the distributions of HDI, a four-quintile category of HDI was defined as follows: low HDI group for HDI < 0.550, medium HDI group for HDI from 0.550 to 0.699, high HDI group for HDI from 0.700 to 0.799, and very high HDI group for HDI ≥0.800.

To analyze the temporal trends of prostate cancer burden, data on the incidence and mortality data between 2000 and 2019 were obtained from the GBD 2019 platform (36). The GBD 2019 platform provided available data for 204 countries or territories, whereas the data quality for each country was spotty (37). As described in GBD 2019 (37), a simple star-rating system from 0 to 5 to was developed to assess the quality of data available in a given country, defined by the percent well-certified data. A country was rated as a 3 star when the percent well-certified data ≥35%, a 4 star for the percent well-certified data ≥65%, and a 5 star for the percent well-certified data ≥85%. To assure the stability and reliability of the temporal trends as previous studies (38, 39), we only analyzed the data for countries with a 3-star or higher level in the GBD dataset, and a total of 89 countries were included in the final analysis (Table 1).

**Table 1:** Estimated incidence and mortality of prostate cancer in the 89 countries in 2020.

**Continent**	**Country**	**HDI in 2019**	**Incidence**			**Mortality**		
			**Cases**	**CR, 1/10^**5**^**	**ASR, 1/10^**5**^**	**Cases**	**CR, 1/10^**5**^**	**ASR, 1/10^**5**^**
World		141,4259	36.0	30.7	375304	9.5	7.7	
Africa	Mauritius	0.804	238	37.9	25.1	110	17.5	11.7
	South Africa	0.709	13152	45	68.3	3896	13.3	22.1
America	Bahamas	0.814	201	105.2	98.0	72.0	37.7	36.3
	Barbados	0.814	279	200.6	110.3	137	98.5	40.3
	Cuba	0.783	6,369	113.3	57.6	3,409	60.6	24.7
	Dominican Republic	0.756	4,808	88.7	88.7	2,228	41.1	35.0
	Jamaica	0.734	1,561	106.2	87.6	844	57.4	39.4
	Saint Lucia	0.759	135	149.3	103.2	54	59.7	32.6
	Trinidad and Tobago	0.796	884	127.9	89.2	403	58.3	38.9
	Belize	0.716	75	37.9	49.1	33	16.7	20.1
	Costa Rica	0.810	1909	75.0	56.6	409	16.1	10.2
	El Salvador	0.673	1365	45.0	42.5	344	11.3	8.4
	Guatemala	0.663	2760	31.3	49.0	786	8.9	12.0
	Mexico	0.779	2,6742	42.4	42.2	7457	11.8	10.6
	Nicaragua	0.66	1,063	32.6	44.7	304	9.3	12.1
	Panama	0.815	1,493	69.1	60.9	406	18.8	13.8
	Argentina	0.845	11,686	53.0	42.0	3,964	18	12.2
	Brazil	0.765	97,278	93.1	78.0	18,345	17.6	13.7
	Chile	0.851	8,157	86.5	56.7	2,96	24.4	14.0
	Colombia	0.767	14,460	57.9	49.8	3,846	15.4	11.9
	Ecuador	0.759	3,249	36.8	35.7	1,272	14.4	12.5
	Guyana	0.682	271	68.5	71.8	90	22.8	21.9
	Paraguay	0.728	1,763	48.6	54	630	17.4	18.3
	Peru	0.777	8,700	53.1	44.3	2,433	14.9	11.4
	Suriname	0.738	154	52.2	57.8	73	24.8	27.1
	Uruguay	0.817	1,544	92.0	60.2	569	33.9	16.8
	Venezuela, Bolivarian Republic of	0.711	7,309	52.3	49.8	3,372	24.1	22.8
	Canada	0.929	29,972	160.0	80.4	4,744	25.3	8.9
	United States of America	0.926	209,512	127.9	72	32,438	19.8	8.2
Asia	China	0.761	115,426	15.6	10.2	51,094	6.9	4.6
	Japan	0.919	106,139	171.9	51.8	13,426	21.7	4.5
	Korea, Republic of	0.916	13,873	54.1	27.3	2,200	8.6	4.1
	Brunei Darussalam	0.838	40	17.6	23.0	8	3.5	5.0
	Philippines	0.718	8,242	15.0	23.4	3,164	5.7	10.8
	Singapore	0.938	1,846	60.3	34.3	371	12.1	7.3
	Thailand	0.777	8,630	25.4	14.6	3,837	11.3	5.9
	Kazakhstan	0.825	1,028	11.3	12.8	532	5.8	6.8
	Kyrgyzstan	0.697	133	4.1	7.5	78	2.4	4.5
	Sri Lanka	0.782	896	8.7	6.3	364	3.5	2.6
	Tajikistan	0.668	80	1.7	3.2	43	0.89	1.8
	Turkmenistan	0.715	118	4	6.3	63	2.1	3.3
	Uzbekistan	0.72	929	5.6	8.5	456	2.7	4.2
	Armenia	0.776	892	64	45.8	346	24.8	16.3
	Azerbaijan	0.756	565	11.2	12.1	218	4.3	5.0
	Bahrain	0.852	51	4.6	13.3	12	1.1	4.5
	Georgia	0.812	1,004	52.8	32.3	457	24	13.4
	Israel	0.919	3,290	76.4	56.1	531	12.3	6.7
	Kuwait	0.806	255	9.8	19.6	52	2	6.6
	Syrian Arab Republic	0.567	1,083	12.4	19.0	422	4.8	7.5
	Turkey	0.82	19,444	46.7	42.5	5,464	13.1	11.3
Europe	Belarus	0.823	4,076	92.6	58.4	1,024	23.3	14.6
	Bulgaria	0.816	4,983	147.7	63.2	1,215	36	13.4
	Czechia	0.9	9,117	172.9	83.0	14,67	27.8	11.0
	Hungary	0.854	6,234	135.6	66.5	1,481	32.2	13.9
	Poland	0.880	18,079	98.6	47.5	7,074	38.6	16.6
	Republic of Moldova	0.75	808	41.8	30.1	368	19	13.9
	Romania	0.828	8,055	86.1	41.5	2,435	26	10.7
	Russian Federation	0.824	46,454	68.7	43.7	14,434	21.3	13.3
	Slovakia	0.860	2,501	94.1	50.8	1,083	40.7	20.9
	Ukraine	0.779	11,361	56.1	33.2	4,219	20.8	11.9
	Denmark	0.94	4,760	165.3	75.6	1,392	48.3	15.9
	Estonia	0.892	1,228	195.4	102.1	331	52.7	21.8
	Finland	0.938	5,710	209	82.1	914	33.5	10.2
	Iceland	0.949	220	128.4	68.3	60	35	13.7
	Ireland	0.955	4,503	183.7	110.7	569	23.2	10.3
	Latvia	0.866	1,531	176.1	87.6	405	46.6	19.9
	Lithuania	0.882	2,237	177.5	94.5	564	44.8	18.4
	Norway	0.957	5,229	190.8	95.6	1,064	38.8	14.3
	Sweden	0.945	10,949	216.4	100.4	2,409	47.6	14.3
	United Kingdom	0.932	56,780	169.3	77.9	13,168	39.3	12.4
	Albania	0.795	794	54.2	28.2	338	23.1	10.6
	Croatia	0.851	2,478	125.2	56.1	812	41	14.7
	Greece	0.888	6,217	121.5	48.2	1,835	35.9	8.2
	Italy	0.892	39,317	133.6	59.9	6,902	23.4	5.9
	Malta	0.895	336	151.7	60.2	53	23.9	8.1
	North Macedonia	0.774	785	75.3	42.6	301	28.9	15.6
	Portugal	0.864	6,759	140.1	59.6	1,917	39.7	10.6
	Serbia	0.806	3,144	73.5	33.4	1,251	29.2	12
	Slovenia	0.917	1,834	177.2	79.3	451	43.6	14.9
	Spain	0.904	34,613	150.6	70.6	5,798	25.2	7.3
	Austria	0.922	6,088	137.1	64.9	1,378	31	10.1
	Belgium	0.931	8,163	142.1	68.1	1,670	29.1	9.2
	France	0.901	66,070	209.2	99	9,060	28.7	8.4
	Germany	0.947	67,959	164.1	66	15,507	37.4	10.6
	Luxembourg	0.916	392	123.8	71.3	63	19.9	8.8
	Switzerland	0.955	6,705	156.2	75.6	1,299	30.3	9.4
	The Netherlands	0.944	14,580	170.8	73.9	2,976	34.9	11.5
Oceania	Australia	0.944	16,973	133.7	72.5	3,455	27.2	10
	New Zealand	0.931	3,938	166.1	92.9	756	31.9	12.3

*CR, crude rate; ASR, age-standardized rate; HDI, human development index*.

### Statistical Analysis

All rates were adjusted by age and presented as age-standardized incidence rates (ASIRs) and age-standardized mortality rates (ASMRs). The standard population used was the Segi's standard population. The correlation between ASIR and ASMR and HDI level was analyzed using generalized additive models applied subject to non-linear associations. Trends in the incidence and mortality rates of prostate cancer were analyzed for each country by using the Joinpoint regression model (40). The average annual percent change (AAPC) was calculated for the full period from 2000 to 2019, and estimated annual percent change (EAPC) was calculated for each segment. The ~95% confidential interval for AAPC and EAPC was also calculated by the empirical quantile method. The 2-tailed *t*-test was used for statistical inference and the null hypothesis of true AAPC or EAPC is 0 and the Bonferroni adjustment was used for multiple tests. A 2-sided *P* < 0.05 was considered to be statistically significant. The Joinpoint regression analyses were conducted with Joinpoint Regression Program version 4.9.0.0 (National Cancer Institute, Bethesda, America) (41).

## Results

### Prostate Cancer Incidence and Mortality in 2020

**Figure 1:**
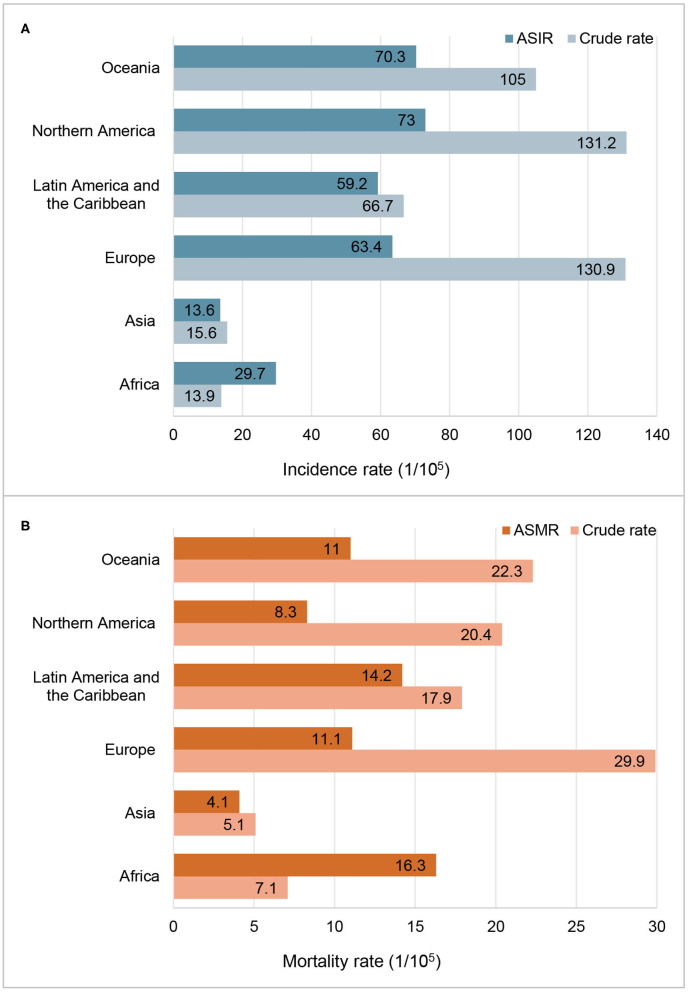
Incidence and mortality of prostate cancer in 2020 by continent. **(A)** Incidence rate; **(B)** Mortality rate; ASIR, age-standardized incidence rate; ASMR, age-standardized mortality rate.

The incidence and mortality of prostate cancer in 2020 of the major countries are shown in Table 1. Globally, more than 1.4 million new prostate cancer cases were diagnosed in 2020. The crude incidence rate was 36.0 per 100,000 males and the ASIR was 30.7 per 100,000 males. Data by continents in Figure 1 showed that, ASIRs in Europe, Latin America and the Caribbean, Northern America and Oceania exceeded 59 per 100,000 males, while ASIRs in Africa and Asia were lower than 30 per 100,000 males. However, the regional distribution of ASMR was quite different, with the highest rate in Africa, followed by Latin America and the Caribbean, Europe, Oceania, Northern America and Asia.

ASIRs substantially vary more than 123-fold among 174 countries, wherein the highest ASIR was 110.7 per 100,000 males in Ireland from Northern Europe while the lowest ASIR was 0.9 per 100,000 males in Bhutan from South-Central Asia. Similarly, ASMRs varied by more than 77-fold among 174 countries, from the lowest ASMR of 0.54 per 100,000 males in Bhutan from South-Central Asia to the highest ASMR of 41.7 per 100,000 males in Zimbabwe from Eastern Africa, in which the crude mortality rate was only 12.2 per 100,000 (Table 1).

**Figure 2:**
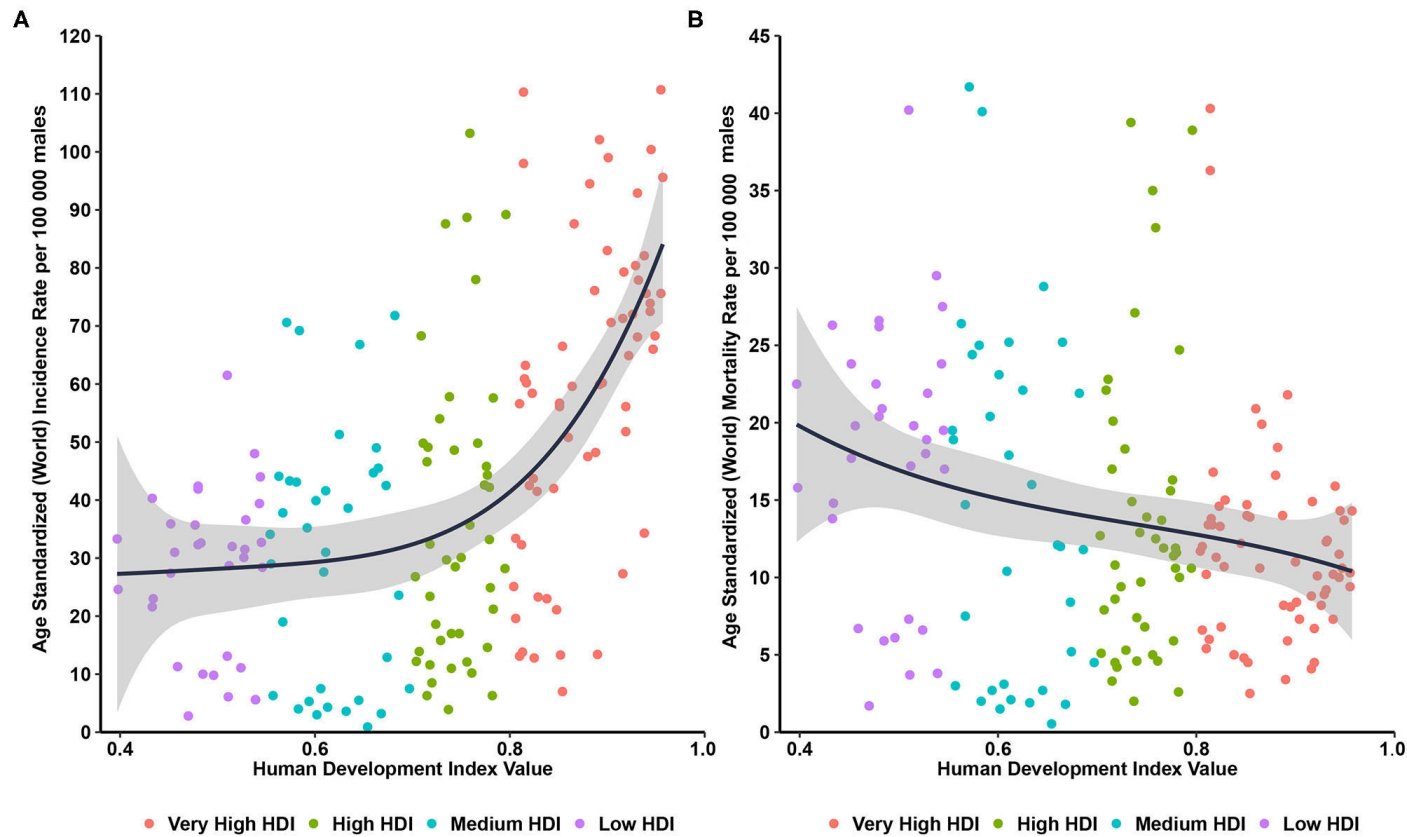
Correlations between age-standardized incidence and mortality of prostate cancer and human development index. **(A)** age-standardized incidence rate; **(B)** age-standardized mortality rate.

Through ecological correlation analysis presented in Figure 2, we found that a positive correlation between the incidence of prostate cancer and HDI (R^2^ = 0.327, *P* < 0.001), with ASIRs in countries with a very high HDI more than twice greater than those in countries with a low HDI, while a negative correlation between the ASMRs of prostate cancer and HDI (R^2^ = 0.05, *P* < 0.001, Figure 2).

### Trend Patterns From 2000 to 2019

By grouping according to the changing trends in the ASIR and ASMR of prostate cancer of 89 countries, six subgroups were divided, including significantly increasing ASIR (*P* < 0.05) and ASMR (*P* < 0.05) in 19 countries (Group A), significantly increasing ASIR (*P* < 0.05) and stable ASMR (*P* ≥ 0.05) in 21 countries (Group B), increasing ASIR (*P* < 0.05) but decreasing ASMR(*P* < 0.05) in 25 countries (Group C), stable ASIR (*P* ≥ 0.05) and ASMR (*P* ≥ 0.05) in 4 countries (Group D), stable ASIR (*P* ≥ 0.05) but decreasing ASMR (*P* < 0.05,) in 11 countries (Group E), and decreasing ASIR (*P* < 0.05) and ASMR (*P* < 0.05) in 9 countries (Group F; Table 2).

**Table 2:** Patterns of trends in incidence and mortality for prostate cancer from 2000 to 2019 among 89 countries.

**Group**	**Incidence^a^**	**Mortality^a^**	**Number of countries**	**List of country (AAPC of incidence, AAPC of mortality, %)**
A	Increasing	Increasing	19	Armenia (2.66, 1), Azerbaijan (1.75, 0.43), Brunei Darussalam (2.74, 1.89), Bulgaria (3.11, 2.17), Croatia (2.26, 0.4), Cuba (1.97, 0.54), Estonia (3.79, 0.48), Georgia (4.04, 3.63), Kazakhstan (2.88, 0.65), Latvia (2.99, 0.97), Lithuania (2.12, 0.52), North Macedonia (2.18, 0.72), Republic of Moldova (4.77, 2.6), Romania (2.81, 0.54), Russian Federation (3.57, 0.87), Serbia (2.63, 0.91), Suriname (1.24, 0.36), Tajikistan (1.37, 0.75), Uzbekistan (1.42, 0.52)
B	Increasing	Stable	21	Albania (1.8, 0.05), Bahrain (1.73, −0.26), Barbados (0.75, −0.17), Belarus (3.21, 0.26), Costa Rica (1.56, −0.19), Dominican Republic (0.69, −0.5), Ecuador (1.52, 0.14), El Salvador (1.67, −0.04), Kuwait (2.34, 0.53), Nicaragua (3.03, 0.26), Paraguay (1.82, 0.1), Philippines (0.58, 0.11), Saint Lucia (0.42, −0.17), Singapore (1.4, −1.14), Slovakia (1.97, −0.08), Sri Lanka (2.48, 0.05), Syrian Arab Republic (1.23, −0.13), Turkey (2.36, −0.72), Turkmenistan (1.64, 0.29), Ukraine (1.56, 0.23), Venezuela, Bolivarian Republic of (1.45, 0.17)
C	Increasing	Decreasing	25	Argentina (0.23, −0.9), Brazil (0.47, −0.98), Chile (1.48, −0.52), China (2.88, −0.59), Czechia (0.61, −0.97), Denmark (1.97, −0.78), Finland (0.29, −1.44), Germany (0.24, −1.41), Guatemala (1.05, −0.42), Hungary (0.43, −1.08), Ireland (0.38, −1.85), Japan (1.46, −0.68), Korea, Republic of (2.63, −0.48), Malta (0.25, −1.76), Mexico (0.99, −0.34), Panama (0.53, −0.81), Peru (1.84, −0.37), Poland (1.41, −0.35), Portugal (0.68, −1.72), Slovenia (2.05, −0.58), South Africa (0.69, −0.32), Thailand (1.14, −0.94), The Netherlands (0.7, −1.06), Trinidad and Tobago (0.31, −0.8), United Kingdom (0.93, −0.73)
D	Stable	Stable	4	Bahamas (0.04, −0.16), Colombia (0.8, −1.31), Jamaica (0.55, 0.23), Kyrgyzstan (0.35, −0.95)
E	Stable	Decreasing	11	Australia (0.04, −1.1), Belgium (−0.11, −1.75), Belize (0.06, −0.91), Greece (-0.2, −1.3), Guyana (0.08, −0.33), Israel (−0.22, −1.99), Italy (−0.11, −1.54), Mauritius (0.23, −0.68), Norway (0.12, −1.3), Spain (−0.06, −1.66), Uruguay (0.29, −0.8)
F	Decreasing	Decreasing	9	Austria (−0.42, −1.73), Canada (−1.5, −2.19), France (−0.29, −2.19), Iceland (−0.63, −1.53), Luxembourg (−0.52, −2.21), New Zealand (−0.43, −1.6), Sweden (−0.48, −1.53), Switzerland (−0.88, −1.69), United States of America (−0.69, −1.22)

a *All rates were adjusted by the Segi's population*.

*AAPC, average annual percentage of change. The terms “increasing” or “decreasing” were used when the AAPC was s tatistically significant (P < 0.05); otherwise, the term “stable” was used*.

**Figure 3:**
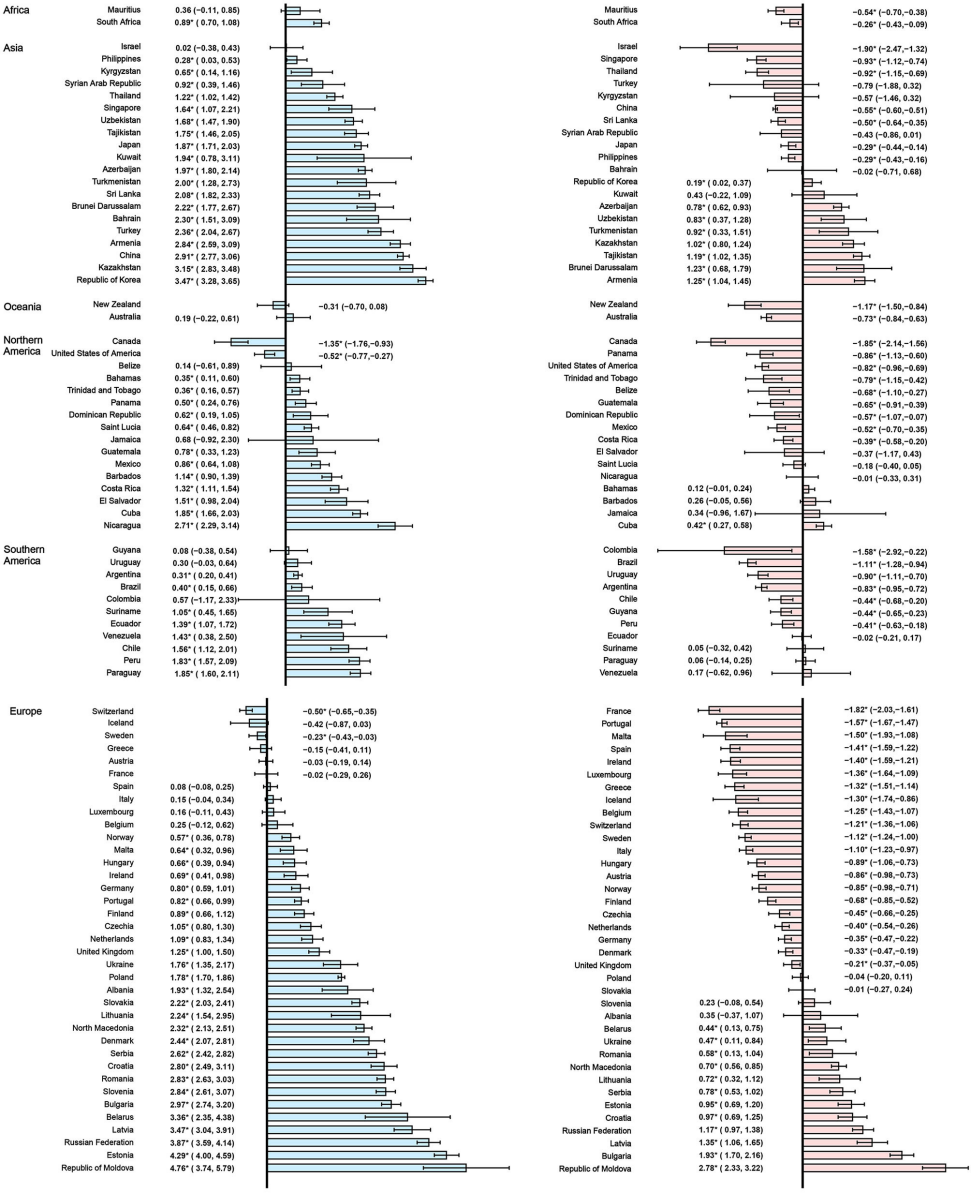
The AAPC of the ASIR (blue bars) and ASMR (red bars) of prostate cancer. *P < 0.05. AAPC, Average annual percent change; ASIR, age-standardized incidence rate; ASMR, age-standardized mortality rate.

In the past two decades, an increasing trend of ASIR for prostate cancer was observed in 65 countries, nearly all countries had high or very high HDI except Tajikistan, and the AAPCs ranged from 0.23% (Argentina) to 4.54% (Republic of Moldova). Meanwhile, significantly increasing mortality trends were also observed in 19 of the 65 countries, with the AAPCs ranging from 0.36% (Suriname) to 3.63% (Georgia) while significantly decreasing mortality trends were also observed in 25 of the 65 countries, with AAPCs ranging from −1.85% (Ireland) to −0.32% (South Africa). In addition, ASIR and ASMR have been significantly decreasing from 2000 to 2019 in nine countries with a very high HDI, including the Austria, Canada, France, Iceland, Luxembourg, New Zealand, Sweden, Switzerland, and United States of America (Tables 3, 4; Figure 3).

**Table 3:** Trend analysis of age-standardized incidence for prostate cancer from 2000 to 2019.

**Country**	**AAPC**	**Range**	**Trend1**			**Trend2**			**Trend3**			**Trend4**		
			**Period**	**EAPC**	**Range**	**Period**	**EAPC**	**Range**	**Period**	**EAPC**	**Range**	**Period**	**EAPC**	**Range**
Albania	1.8^*^	1.17–2.42	2000–13	1.44^*^	1.19–1.69	2013–16	4.06^*^	0.17–8.1	2016–19	1.12	−0.65–2.92			
Argentina	0.23^*^	0.08–0.38	2000–06	1.09^*^	0.77–1.4	2006–15	−0.94^*^	−1.13 to −0.76	2015–19	1.61^*^	1.1–2.12			
Armenia	2.66^*^	2.44–2.88	2000–11	3.24^*^	2.93–3.54	2011–19	1.86^*^	1.47–2.26						
Australia	0.04	−0.38–0.46	2000–04	4.12^*^	2.99–5.27	2004–08	0.58	−0.97–2.15	2008–14	−2.94^*^	−3.58 to −2.29	2014–19	0.04	−0.6–0.68
Austria	−0.42^*^	−0.58 to −0.26	2000–04	0.24	−0.35–0.82	2004–12	−1.07^*^	−1.3 to −0.83	2012–19	−0.05	−0.28–0.18			
Azerbaijan	1.75^*^	1.49–2.02	2000–09	2.39^*^	2.12–2.66	2009–17	1.56^*^	1.24–1.89	2017–19	−0.35	−2.45–1.79			
Bahamas	0.04	−0.08–0.17	2000–19	0.04	−0.08–0.17									
Bahrain	1.73^*^	0.93–2.54	2000–03	7.82^*^	3.97–11.8	2003–09	1.3	−0.03–2.66	2009–13	−2.96^*^	−5.41 to −0.46	2013–19	2.42^*^	1.75–3.1
Barbados	0.75^*^	0.53–0.96	2000–04	1.1^*^	0.39–1.81	2004–17	−0.28^*^	−0.39 to −0.16	2017–19	6.89^*^	5.15–8.65			
Belarus	3.21^*^	2.25–4.19	2000–05	1.94^*^	0.09–3.83	2005–10	5.56^*^	3.06–8.12	2010–17	3.37^*^	2.23–4.52	2017–19	0.11	−5.57–6.14
Belgium	−0.11	−0.48–0.26	2000–04	4.52^*^	3.6–5.45	2004–08	0.06	−1.24–1.39	2008–13	−4.54^*^	−5.35 to −3.73	2013–19	0.54^*^	0.09–0.99
Belize	0.06	−0.71–0.83	2000–03	−1.54	−3.76–0.74	2003–06	2.14	−2.17–6.64	2006–10	−2.14^*^	−4.09 to −0.15	2010–19	0.9^*^	0.58–1.21
Brazil	0.47^*^	0.2–0.73	2000–04	2.28^*^	1.73–2.82	2004–07	0.65	−0.88–2.2	2007–17	−0.57^*^	−0.7 to −0.45	2017–19	1.85^*^	0.61–3.1
Brunei Darussalam	2.74^*^	2.24–3.25	2000–05	6.19^*^	4.62–7.78	2005–13	2.71^*^	1.99–3.43	2013–19	0	−0.77–0.77			
Bulgaria	3.11^*^	2.89–3.34	2000–06	5.65^*^	5.33–5.96	2006–10	3.78^*^	2.99–4.57	2010–14	2.58^*^	1.85–3.3	2014–19	0.06	−0.24–0.36
Canada	−1.5^*^	−1.91 to −1.08	2000–03	0.43	−0.84–1.72	2003–11	−3.85^*^	−4.17 to −3.53	2011–14	−2.42	−4.85–0.08	2014–19	1.78^*^	1.24–2.31
Chile	1.48^*^	1.05–1.91	2000–04	2.89^*^	2.03–3.76	2004–07	0.71	−1.69–3.17	2007–11	2.74^*^	1.61–3.88	2011–19	0.45^*^	0.24–0.66
China	2.88^*^	2.57–3.18	2000–07	3.27^*^	2.97–3.57	2007–10	4.41^*^	2.54–6.32	2010–16	1.79^*^	1.44–2.15	2016–19	2.63^*^	1.94–3.32
Colombia	0.8	−0.94–2.58	2000–02	5.61	−4.82–17.18	2002–05	−4.23	−13.11–5.56	2005–16	0.7^*^	0.01–1.39	2016–19	3.25	−0.46–7.1
Costa Rica	1.56^*^	1.35–1.78	2000–19	1.56^*^	1.35–1.78									
Croatia	2.26^*^	1.96–2.56	2000–05	4.92^*^	4.06–5.79	2005–15	1.89^*^	1.6–2.19	2015–19	−0.08	−0.95–0.81			
Cuba	1.97^*^	1.78–2.15	2000–06	2.85^*^	2.29–3.42	2006–19	1.56^*^	1.42–1.7						
Czechia	0.61^*^	0.37–0.85	2000–04	3.33^*^	2.62–4.05	2004–09	1.86^*^	1.24–2.49	2009–15	−1.76	−2.16 to −1.36	2015–19	−0.04	−0.6–0.52
Denmark	1.97^*^	1.59–2.36	2000–04	8.26^*^	7.07–9.46	2004–09	4.22^*^	3.27–5.17	2009–14	−2.6	−3.42 to −1.77	2014–19	−0.42	−1.01–0.18
Dominican Republic	0.69^*^	0.3–1.09	2000–06	2.78^*^	2.35–3.21	2006–09	−2.56^*^	−4.73 to −0.33	2009–13	2.15^*^	1.07–3.25	2013–19	−0.68^*^	−1.01 to −0.34
Ecuador	1.52^*^	1.19–1.85	2000–07	3.54^*^	2.68–4.41	2007–19	0.36^*^	0.07–0.64						
El Salvador	1.67^*^	0.78–2.57	2000–02	0.18	−6.07–6.86	2002–06	4.9^*^	1.84–8.06	2006–13	−1.32^*^	−2.24 to −0.39	2013–19	3.6^*^	2.74–4.47
Estonia	3.79^*^	3.51–4.08	2000–08	7.08^*^	6.65–7.51	2008–15	2.79^*^	2.3–3.29	2015–19	−0.81	−1.64–0.03			
Finland	0.29^*^	0.09–0.48	2000–04	2.92^*^	2.18–3.68	2004–14	−0.86^*^	−1.05 to −0.68	2014–19	0.51^*^	0.08–0.95			
France	−0.29^*^	−0.56 to −0.02	2000–03	3.84^*^	2.78–4.91	2003–07	−0.24	−1.21–0.73	2007–13	−3.08^*^	−3.5 to −2.66	2013–19	0.49^*^	0.16–0.81
Georgia	4.04^*^	3.56–4.53	2000–05	10.84^*^	9.41–12.29	2005–12	3.22^*^	2.35–4.1	2012–19	0.23	−0.4–0.86			
Germany	0.24^*^	0.02–0.46	2000–04	2.45^*^	2.01–2.88	2004–09	0.47^*^	0.07–0.87	2009–12	−1.85^*^	−3.07 to −0.61	2012–19	−0.27^*^	−0.43 to −0.1
Greece	−0.2	−0.45–0.05	2000–04	1.56^*^	0.76–2.36	2004–16	−1.11^*^	−1.28 to −0.95	2016–19	1.16	−0.1–2.44			
Guatemala	1.05^*^	0.61–1.49	2000–05	3.72^*^	2.78–4.67	2005–12	0.57^*^	0.01–1.14	2012–17	−1.32^*^	−2.26 to −0.37	2017–19	2.11	−0.74–5.05
Guyana	0.08	−0.21–0.36	2000–06	−1.31^*^	−1.86 to −0.76	2006–12	0.18	−0.52–0.88	2012–19	1.2^*^	0.81–1.58			
Hungary	0.43^*^	0.15–0.7	2000–05	0.86^*^	0.15–1.57	2005–12	−0.61^*^	−1.12 to −0.1	2012–19	1.16^*^	0.77–1.55			
Iceland	−0.63^*^	−1.08 to −0.17	2000–07	1.08^*^	0.69–1.47	2007–12	−1.34^*^	−2.18 to −0.5	2012–15	−4.28^*^	−6.8 to −1.68	2015–19	0.1	−0.71–0.91
Ireland	0.38^*^	0.1–0.65	2000–04	3.75^*^	3.02–4.49	2004–09	0.33	−0.31–0.97	2009–13	−2.95^*^	−3.88 to −2	2013–19	0.46^*^	0.15–0.76
Israel	−0.22	−0.6–0.17	2000–08	1.82^*^	1.3–2.34	2008–14	−3.38^*^	−4.27 to −2.47	2014–19	0.41	−0.46–1.29			
Italy	−0.11	−0.3–0.08	2000–03	3.22^*^	2.48–3.95	2003–07	−0.04	−0.71–0.64	2007–13	−1.89^*^	−2.19 to −1.6	2013–19	0.01	−0.22–0.24
Jamaica	0.55	−1.05–2.18	2000–06	1.06	−0.77–2.92	2006–09	8.21	−1.53–18.91	2009–14	−4.68^*^	−7.47 to −1.8	2014–19	0.88	−1.14–2.95
Japan	1.46^*^	1.3–1.62	2000–05	5.95^*^	5.59–6.32	2005–09	1.91^*^	1.24–2.58	2009–19	−0.89^*^	−0.98 to −0.79			
Kazakhstan	2.88^*^	2.68–3.08	2000–11	3.29^*^	3.21–3.36	2011–14	2.08^*^	1.11–3.05	2014–17	4.44^*^	3.53–5.35	2017–19	−0.42	−1.21–0.37
Kuwait	2.34^*^	1.19–3.5	2000–03	0.1	−3.74–4.09	2003–08	5.49^*^	3.21–7.82	2008–11	−3.6	−9.3–2.45	2011–19	3.55^*^	3–4.11
Kyrgyzstan	0.35	−0.12–0.82	2000–04	−3.06^*^	−3.94 to −2.16	2004–12	0.9^*^	0.49–1.31	2012–15	2.69	−0.16–5.63	2015–19	0.97^*^	0.17–1.78
Latvia	2.99^*^	2.54–3.44	2000–07	6.92^*^	6.52–7.33	2007–11	0.38	−0.84–1.63	2011–14	2.94^*^	0.49–5.44	2014–19	−0.22	−0.74–0.3
Lithuania	2.12^*^	1.47–2.78	2000–04	6.41^*^	4.86–7.98	2004–07	10.16^*^	5.87–14.61	2007–19	−1.16^*^	−1.38 to −0.94			
Luxembourg	−0.52^*^	−0.79 to −0.24	2000–04	1.76^*^	1.06–2.46	2004–08	−0.56	−1.58–0.47	2008–16	−1.99^*^	−2.25 to −1.73	2016–19	0.5	−0.43–1.44
Malta	0.25^*^	0.04–0.46	2000–09	1.25^*^	1.01–1.48	2009–15	−1.48^*^	−1.95 to −1	2015–19	0.62	−0.03–1.28			
Mauritius	0.23^*^	−0.34–0.79	2000–02	2.71	−1.2–6.77	2002–06	−1.8	−3.61–0.03	2006–17	0.22	−0.04–0.47	2017–19	1.95	−0.81–4.78
Mexico	0.99^*^	0.78–1.21	2000–05	1.05^*^	0.44–1.66	2005–16	0.44^*^	0.27–0.62	2016–19	2.95^*^	1.98–3.93			
The Netherlands	0.7^*^	0.45–0.94	2000–03	4.6^*^	3.2–6.02	2003–14	−0.25^*^	−0.44 to −0.07	2014–19	0.51^*^	0.03–0.99			
New Zealand	−0.43^*^	−0.81 to −0.05	2000–02	0.09	−2.07–2.31	2002–05	−2.94^*^	−5.03 to −0.8	2005–13	−1.08^*^	−1.36 to −0.8	2013–19	1.55^*^	1.23–1.88
Nicaragua	3.03^*^	2.61–3.45	2000–12	3.77^*^	3.56–3.97	2012–15	−0.04	−2.59–2.58	2015–19	3.15^*^	2.39–3.92			
North Macedonia	2.18^*^	1.97–2.4	2000–05	3.02^*^	2.61–3.44	2005–09	4.07^*^	3.25–4.9	2009–16	1.59^*^	1.35–1.83	2016–19	−0.27	−0.9–0.36
Norway	0.12	−0.09–0.33	2000–09	2.05^*^	1.68–2.42	2009–19	−1.58^*^	−1.85 to −1.32						
Panama	0.53^*^	0.28–0.79	2000–13	−0.41^*^	−0.67 to −0.16	2013–19	2.62^*^	1.93–3.31						
Paraguay	1.82^*^	1.56–2.08	2000–04	2.53^*^	1.51–3.56	2004–13	0.62^*^	0.32–0.92	2013–19	3.17^*^	2.78–3.56			
Peru	1.84^*^	1.51–2.18	2000–04	2.15^*^	1.56–2.74	2004–07	0.49	−1.16–2.17	2007–10	4.17^*^	2.59–5.78	2010–19	1.39^*^	1.27–1.51
Philippines	0.58^*^	0.34–0.81	2000–07	−0.75^*^	−1.14 to −0.36	2007–13	2.51^*^	1.92–3.1	2013–19	0.23	−0.15–0.6			
Poland	1.41^*^	1.32–1.49	2000–09	2.52^*^	2.43–2.62	2009–15	0.93^*^	0.72–1.13	2015–19	−0.35^*^	−0.61 to −0.1			
Portugal	0.68^*^	0.51–0.85	2000–04	1.75^*^	1.14–2.37	2004–13	−0.25^*^	−0.45 to −0.05	2013–19	1.37^*^	1.08–1.67			
Korea, Republic of	2.63^*^	2.46–2.8	2000–04	7.24^*^	6.51–7.96	2004–10	4.48^*^	4.11–4.85	2010–19	−0.54^*^	−0.66 to −0.43			
Republic of Moldova	4.77^*^	4.35–5.2	2000–16	5.97^*^	5.7–6.24	2016–19	−1.37	−3.77–1.09						
Romania	2.81^*^	2.61–3.01	2000–12	3.44^*^	3.21–3.67	2012–19	1.74^*^	1.31–2.17						
Russian Federation	3.57^*^	3.28–3.86	2000–15	4.67^*^	4.45–4.89	2015–19	−0.46	−1.66–0.76						
Saint Lucia	0.42^*^	0.24–0.6	2000–11	−0.5^*^	−0.74 to −0.26	2011–19	1.71^*^	1.38–2.03						
Serbia	2.63^*^	2.45–2.82	2000–08	5.06^*^	4.79–5.32	2008–15	1.69^*^	1.36–2.02	2015–19	−0.45	−1.02–0.12			
Singapore	1.4^*^	1.17–1.62	2000–09	3.07^*^	2.64–3.51	2009–19	−0.09	−0.34–0.16						
Slovakia	1.97^*^	1.78–2.17	2000–04	1.27^*^	0.69–1.84	2004–09	4.93^*^	4.4–5.47	2009–15	2.11^*^	1.79–2.42	2015–19	−1.12^*^	−1.51 to −0.73
Slovenia	2.05^*^	1.82–2.28	2000–09	4.13^*^	3.71–4.56	2009–19	0.21	−0.07–0.49						
South Africa	0.69^*^	0.53–0.86	2000–05	−0.39^*^	−0.72 to −0.06	2005–10	2.56^*^	2.12–3	2010–16	1.59^*^	1.32–1.86	2016–19	−2.32^*^	−2.85 to −1.78
Spain	−0.06	−0.22–0.11	2000–03	2.86^*^	2.2–3.53	2003–09	−0.35^*^	−0.62 to −0.07	2009–13	−1.95^*^	−2.54 to −1.36	2013–19	0.07	−0.13–0.27
Sri Lanka	2.48^*^	2.2–2.76	2000–04	2.44^*^	1.32–3.56	2004–12	3.56^*^	3.16–3.96	2012–19	1.28^*^	0.97–1.59			
Suriname	1.24^*^	0.69–1.8	2000–02	3.96	−0.56–8.68	2002–09	−0.02	−0.7–0.67	2009–14	2.25^*^	1.1–3.41	2014–19	0.95^*^	0.24–1.66
Sweden	−0.48^*^	−0.68 to −0.28	2000–04	2.09^*^	1.42–2.77	2004–16	−1.45^*^	−1.58 to −1.32	2016–19	0.05	−0.93–1.03			
Switzerland	−0.88^*^	−1.02 to −0.73	2000–04	1.18^*^	0.79–1.57	2004–09	−1.53^*^	−1.89 to −1.16	2009–15	−2.33^*^	−2.58 to −2.08	2015–19	0.1	−0.26–0.45
Syrian Arab Republic	1.23^*^	0.68–1.79	2000–04	−1.85^*^	−3.14 to −0.53	2004–12	3.3^*^	2.81–3.8	2012–15	−0.39^*^	−3.45–2.76	2015–19	1.49^*^	0.55–2.44
Tajikistan	1.37^*^	1.05–1.69	2000–16	1.75^*^	1.58–1.93	2016–19	−0.67	−2.61–1.31						
Thailand	1.14^*^	0.95–1.33	2000–13	0.73^*^	0.53–0.93	2013–19	2.04^*^	1.55–2.53						
Trinidad and Tobago	0.31^*^	0.1–0.52	2000–08	0.45^*^	0.19–0.72	2008–13	−0.71^*^	−1.38 to −0.03	2013–19	0.96^*^	0.64–1.29			
Turkey	2.36^*^	1.97–2.74	2000–04	9.74^*^	8.45–11.05	2004–11	0.77^*^	0.25–1.29	2011–16	−0.73	−1.58–0.13	2016–19	1.81^*^	0.54–3.09
Turkmenistan	1.64^*^	0.99–2.29	2000–02	−1.29	−5.44–3.04	2002–06	4.1^*^	2.05–6.2	2006–10	−0.15	−1.96–1.69	2010–19	2.01^*^	1.73–2.3
Ukraine	1.56^*^	1.18–1.94	2000–06	−0.53	−1.62–0.57	2006–19	2.53^*^	2.21–2.86						
United Kingdom	0.93^*^	0.67–1.19	2000–02	3.16^*^	1.56–4.79	2002–09	1.04^*^	0.79–1.28	2009–12	−0.02	−1.38–1.37	2012–19	0.6^*^	0.43–0.78
United States of America	−0.69^*^	−0.92 to −0.46	2000–02	−1	−2.56–0.6	2002–06	−1.92^*^	−2.69 to −1.14	2006–15	−0.59^*^	−0.75 to −0.43	2015–19	0.49^*^	0.06–0.91
Uruguay	0.29	−0.05–0.62	2000–04	2.99^*^	2.09–3.89	2004–10	0.14	−0.45–0.73	2010–15	−2.34^*^	−3.15 to −1.52	2015–19	1.17^*^	0.35–2.01
Uzbekistan	1.42^*^	0.99–1.85	2000–02	3.57^*^	0.95–6.26	2002–12	1.4^*^	1.17–1.63	2012–15	0.02	−2.24–2.33	2015–19	1.46^*^	0.77–2.15
Venezuela, Bolivarian Republic of	1.45^*^	0.4–2.5	2000–08	4.63^*^	3.06–6.24	2008–15	−2.98^*^	−4.8 to −1.12	2015–19	3.1	−0.05–6.35			

* *p < 0.05*.

*AAPC, Average Annual Percentage Change; EAPC, Estimated Annual Percentage Change*.

**Table 4:** Trend analysis of age-standardized mortality for prostate cancer from 2000 to 2019.

**Country**	**AAPC**	**Range**	**Trend1**			**Trend2**			**Trend3**			**Trend4**		
			**Period**	**EAPC**	**Range**	**Period**	**EAPC**	**Range**	**Period**	**EAPC**	**Range**	**Period**	**EAPC**	**Range**
Albania	0.05	−0.47–0.57	2000–04	0.81	−0.67–2.32	2004–08	−2.72^*^	−4.86 to −0.54	2008–19	0.8^*^	0.54–1.06			
Argentina	−0.9^*^	−1.01 to −0.78	2000–03	0.09	−0.34–0.52	2003–07	−0.9^*^	−1.31 to −0.48	2007–14	−1.8^*^	−1.94 to −1.67	2014–19	−0.2^*^	−0.37 to −0.02
Armenia	1^*^	0.76–1.25	2000–16	1.32^*^	1.18–1.46	2016–19	−0.65	−2.14–0.87						
Australia	−1.1^*^	−1.2 to −0.99	2000–03	0.38	−0.06–0.82	2003–08	−1.35^*^	−1.61 to −1.09	2008–14	−2.99^*^	−3.17 to −2.82	2014–19	0.59^*^	0.42–0.76
Austria	−1.73^*^	−1.87 to −1.6	2000–04	−3.09^*^	−3.55 to −2.61	2004–14	−1.77^*^	−1.91 to −1.64	2014–19	−0.55^*^	−0.88 to −0.23			
Azerbaijan	0.43^*^	0.29–0.58	2000–03	2.83^*^	2.12–3.55	2003–17	0.27^*^	0.21–0.33	2017–19	−1.94^*^	−2.97 to −0.89			
Bahamas	−0.16	−0.9–0.58	2000–02	3.3	−1.19–8	2002–05	−2.58	−6.6–1.61	2005–19	−0.12	−0.29–0.05			
Bahrain	−0.26	−1–0.49	2000–04	6.02^*^	2.91–9.22	2004–14	−3.04^*^	−3.75 to −2.32	2014–19	0.51	−0.94–1.98			
Barbados	−0.17	−0.51–0.16	2000–04	1.38^*^	0.53–2.24	2004–12	−2.61^*^	−2.94 to −2.27	2012–17	0.17	−0.61–0.95	2017–19	5.91^*^	3.6–8.27
Belarus	0.26	−0.03–0.54	2000–10	0.9^*^	0.48–1.32	2010–19	−0.45	−0.91–0.01						
Belgium	−1.75^*^	−1.97 to −1.53	2000–07	−1.61^*^	−1.78 to −1.44	2007–13	−3.65^*^	−3.93 to −3.36	2013–16	−1.17	−2.47–0.15	2016–19	1.24^*^	0.6–1.88
Belize	−0.91^*^	−1.35 to −0.48	2000–03	−2.27^*^	−3.5 to −1.04	2003–06	−0.5	−2.9–1.95	2006–10	−3.28^*^	−4.41 to −2.13	2010–19	0.48^*^	0.29–0.66
Brazil	−0.98^*^	−1.14 to −0.83	2000–04	0.25	−0.18–0.68	2004–09	−1.1^*^	−1.49 to −0.71	2009–14	−2^*^	−2.37 to −1.63	2014–19	−0.82^*^	−1.06 to −0.57
Brunei Darussalam	1.89^*^	1.47–2.31	2000–13	3.43^*^	3–3.85	2013–19	−1.35^*^	−2.43 to −0.26						
Bulgaria	2.17^*^	1.94–2.41	2000–08	4.68^*^	4.38–4.99	2008–13	1.87^*^	1.13–2.61	2013–19	−0.84^*^	−1.21 to −0.46			
Canada	−2.19^*^	−2.65 to −1.74	2000–04	−2^*^	−2.86 to −1.14	2004–12	−4.63^*^	−4.99 to −4.27	2012–15	−1.06	−3.81–1.77	2015–19	1.79^*^	0.95–2.63
Chile	−0.52^*^	−0.78 to −0.27	2000–12	−0.33^*^	−0.47 to −0.19	2012–16	−1.55^*^	−2.58 to −0.51	2016–19	0.1	−0.89–1.1			
China	−0.59^*^	−0.69 to −0.5	2000–08	−0.82^*^	−1.01 to −0.62	2008–19	−0.43^*^	−0.53 to −0.33						
Colombia	−1.31^*^	−2.65–0.05	2000–02	3.77	−4.23–12.43	2002–05	−5.74	−12.68–1.75	2005–15	−1.87^*^	−2.54 to −1.21	2015–19	1.05	−1.03–3.19
Costa Rica	−0.19	−0.38–0.01	2000–19	−0.19	−0.38–0.01									
Croatia	0.4^*^	0.14–0.66	2000–05	2.38^*^	1.65–3.12	2005–15	−0.04	−0.3–0.21	2015–19	−0.94^*^	−1.75 to −0.12			
Cuba	0.54^*^	0.39–0.69	2000–09	0.8^*^	0.53–1.07	2009–19	0.3^*^	0.11–0.5						
Czechia	−0.97^*^	−1.16 to −0.78	2000–04	0.33	−0.2–0.87	2004–10	−1.17^*^	−1.52 to −0.82	2010–15	−2.3^*^	−2.77 to −1.83	2015–19	−0.28	−0.75–0.19
Denmark	−0.78^*^	−0.94 to −0.62	2000–05	1.3^*^	1.03–1.58	2005–09	−0.77^*^	−1.34 to −0.19	2009–14	−3.12^*^	−3.47 to −2.77	2014–19	−0.48^*^	−0.73 to −0.24
Dominican Republic	−0.5	−1.04–0.05	2000–05	2.16^*^	1.36–2.96	2005–08	−5.12^*^	−8.22 to −1.92	2008–15	0.91^*^	0.37–1.45	2015–19	−2.65^*^	−3.57 to −1.72
Ecuador	0.14	−0.02–0.31	2000–06	1.88^*^	1.53–2.22	2006–12	−0.23	−0.62–0.15	2012–19	−1^*^	−1.2 to −0.79			
El Salvador	−0.04	−0.86–0.78	2000–02	−2.21	−7.76–3.68	2002–06	2.81^*^	0.01–5.69	2006–13	−2.65^*^	−3.52 to −1.76	2013–19	1.91^*^	1.06–2.78
Estonia	0.48^*^	0.24–0.72	2000–06	2.55^*^	2.04–3.07	2006–15	0.11	−0.17–0.4	2015–19	−1.74^*^	−2.51 to −0.97			
Finland	−1.44^*^	−1.6 to −1.28	2000–02	−1.05	−2.27–0.18	2002–08	−3.07^*^	−3.33 to −2.8	2008–14	−1.3^*^	−1.56 to −1.04	2014–19	0.22	−0.02–0.47
France	−2.19^*^	−2.39 to −2	2000–03	−2.02^*^	−2.58 to −1.46	2003–11	−3.75^*^	−3.9 to −3.6	2011–14	−3.02^*^	−4.18 to −1.84	2014–19	0.75^*^	0.5–1.01
Georgia	3.63^*^	3.19–4.07	2000–05	10.27^*^	8.97–11.6	2005–12	3.02^*^	2.24–3.82	2012–19	−0.3	−0.87–0.28			
Germany	−1.41^*^	−1.53 to −1.28	2000–08	−1.97^*^	−2.04 to −1.89	2008–11	−2.31^*^	−2.99 to −1.62	2011–15	−1.08^*^	−1.43 to −0.73	2015–19	0.08	−0.14–0.3
Greece	−1.3^*^	−1.49 to −1.11	2000–03	−1.03	−1.74 to −0.31	2003–11	−3.16^*^	−3.35 to −2.97	2011–16	−0.16	−0.64–0.31	2016–19	1.58^*^	0.85–2.32
Guatemala	−0.42^*^	−0.7 to −0.13	2000–05	1.32^*^	0.86–1.79	2005–14	−1.1^*^	−1.28 to −0.91	2014–17	−2.02^*^	−3.56 to −0.46	2017–19	0.79	−0.7–2.31
Guyana	−0.33	−0.65–0	2000–04	−1.38^*^	−2.05 to −0.71	2004–11	−0.14	−0.48–0.21	2011–14	1.13	−0.82–3.11	2014–19	−0.61^*^	−1.02 to −0.19
Hungary	−1.08^*^	−1.23 to −0.93	2000–13	−1.64^*^	−1.77 to −1.51	2013–19	0.13	−0.3–0.57						
Iceland	−1.53^*^	−1.94 to −1.11	2000–11	−0.81^*^	−1 to −0.62	2011–14	−5.3^*^	−7.83 to −2.7	2014–19	−0.81^*^	−1.39 to −0.21			
Ireland	−1.85^*^	−2.05 to −1.64	2000–04	−1.69^*^	−2.08 to −1.3	2004–14	−2.73^*^	−2.84 to −2.62	2014–17	−0.64	−1.8–0.55	2017–19	0.49	−0.63–1.63
Israel	−1.99^*^	−2.51 to −1.46	2000–09	−1.93^*^	−2.2 to −1.66	2009–13	−4.47^*^	−5.89 to −3.03	2013–16	−1.32	−4.19–1.63	2016–19	0.54	−0.84–1.95
Italy	−1.54^*^	−1.63 to −1.45	2000–03	−1.61^*^	−2.08 to −1.14	2003–14	−2.25^*^	−2.32 to −2.18	2014–19	0.07	−0.14–0.28			
Jamaica	0.23	−1.07–1.55	2000–06	−0.49	−1.85–0.89	2006–09	8.46^*^	0.79–16.72	2009–13	−5.07^*^	−8.5 to −1.51	2013–19	0.64	−0.56–1.85
Japan	−0.68^*^	−0.81 to −0.54	2000–05	−0.38^*^	−0.57 to −0.19	2005–08	−1.47^*^	−2.24 to −0.68	2008–17	−0.79^*^	−0.87 to −0.71	2017–19	0.28	−0.45–1.01
Kazakhstan	0.65^*^	0.22–1.09	2000–11	1.35^*^	1.19–1.5	2011–14	−0.73	−2.72–1.3	2014–17	1.24	−0.75–3.27	2017–19	−1.91^*^	−3.73 to −0.06
Kuwait	0.53	−0.13–1.19	2000–08	2.12^*^	1.57–2.67	2008–11	−5.23^*^	−9.19 to −1.09	2011–19	1.17^*^	0.76–1.58			
Kyrgyzstan	−0.95	−1.97–0.08	2000–03	−4.44^*^	−7.26 to −1.54	2003–13	−1^*^	−1.59 to −0.42	2013–16	3.29	−2.74–9.69	2016–19	−1.35	−4.12–1.51
Latvia	0.97^*^	0.7–1.23	2000–06	5.14^*^	4.56–5.72	2006–14	−0.28	−0.67–0.1	2014–19	−1.89^*^	−2.53 to −1.26			
Lithuania	0.52^*^	0.14–0.91	2000–07	5.43^*^	4.91–5.94	2007–11	−3.03^*^	−4.62 to −1.42	2011–19	−1.83^*^	−2.2 to −1.46			
Luxembourg	−2.21^*^	−2.54 to −1.88	2000–02	−4.37^*^	−6.55 to −2.14	2002–06	−1.92^*^	−3.05 to −0.77	2006–15	−3.09^*^	−3.33 to −2.85	2015–19	0.61	−0.07–1.29
Malta	−1.76^*^	−2.17 to −1.35	2000–13	−1.62^*^	−1.77 to −1.48	2013–16	−4.42^*^	−6.86 to −1.92	2016–19	0.35	−0.89–1.6			
Mauritius	−0.68^*^	−0.83 to −0.53	2000–19	−0.68^*^	−0.83 to −0.53									
Mexico	−0.34^*^	−0.52 to −0.16	2000–16	−0.51^*^	−0.61 to −0.41	2016–19	0.59	−0.53–1.71						
The Netherlands	−1.06^*^	−1.34 to −0.77	2000–03	0.16	−0.7–1.03	2003–06	−3.6^*^	−5.24 to −1.93	2006–13	−1.54^*^	−1.82 to −1.26	2013–19	0.19	−0.07–0.45
New Zealand	−1.6^*^	−1.92 to −1.28	2000–05	−3.38^*^	−3.93 to −2.83	2005–12	−2.08^*^	−2.5 to −1.66	2012–17	−0.44	−1.18–0.32	2017–19	1.74	−0.48–4.01
Nicaragua	0.26	−0.02–0.54	2000–05	1.77^*^	1.22–2.32	2005–12	−0.27^*^	−0.63–0.09	2012–16	−1.78^*^	−2.76 to −0.79	2016–19	1.79^*^	0.84–2.75
North Macedonia	0.72^*^	0.53–0.9	2000–07	1.4^*^	1.1–1.71	2007–16	0.81^*^	0.6–1.03	2016–19	−1.16^*^	−2.04 to −0.27			
Norway	−1.3^*^	−1.44 to −1.16	2000–07	−1.06^*^	−1.23 to −0.88	2007–17	−1.7^*^	−1.82 to −1.59	2017–19	−0.16	−1.37–1.06			
Panama	−0.81^*^	−1.1 to −0.52	2000–03	0.9	−0.71–2.54	2003–11	−1.48^*^	−1.86 to −1.09	2011–19	−0.77^*^	−1.05 to −0.49			
Paraguay	0.1	−0.19–0.39	2000–04	1.59^*^	0.81–2.37	2004–10	−0.51^*^	−1 to −0.02	2010–14	−1.21^*^	−2.23 to −0.18	2014–19	0.69^*^	0.25–1.12
Peru	−0.37^*^	−0.59 to −0.16	2000–07	−0.9^*^	−1.09 to −0.71	2007–10	2.83^*^	1.51–4.18	2010–15	−1.62^*^	−1.99 to −1.24	2015–19	−0.26	−0.61–0.1
Philippines	0.11	−0.02–0.25	2000–04	−1.47^*^	−1.85 to −1.09	2004–09	0.71^*^	0.35–1.07	2009–15	1.1^*^	0.87–1.33	2015–19	−0.5^*^	−0.79 to −0.21
Poland	−0.35^*^	−0.52 to −0.17	2000–06	−0.07	−0.39–0.24	2006–11	−1.06^*^	−1.61 to −0.5	2011–19	−0.11	−0.28–0.07			
Portugal	−1.72^*^	−1.83 to −1.62	2000–02	−3.24^*^	−3.99 to −2.49	2002–07	−2.53^*^	−2.77 to −2.29	2007–14	−2.11^*^	−2.24 to −1.98	2014–19	0.27^*^	0.1–0.44
Korea, Republic of	−0.48^*^	−0.67 to −0.28	2000–08	0.63^*^	0.47–0.78	2008–11	−0.31	−1.44–0.85	2011–16	−2.07^*^	−2.39 to −1.74	2016–19	−0.9^*^	−1.37 to −0.43
Republic of Moldova	2.6^*^	2.2–3	2000–15	3.82^*^	3.53–4.11	2015–19	−1.86^*^	−3.54 to −0.15						
Romania	0.54^*^	0.27–0.8	2000–07	0.19	−0.23–0.61	2007–15	1.2^*^	0.8–1.59	2015–19	−0.17	−1.02–0.69			
Russian Federation	0.87^*^	0.68–1.05	2000–15	1.54^*^	1.41–1.67	2015–19	−1.63^*^	−2.45 to −0.8						
Saint Lucia	−0.17	−0.62–0.27	2000–02	0.84	−3.17–5.02	2002–11	−1.93^*^	−2.34 to −1.53	2011–19	1.59^*^	1.23–1.95			
Serbia	0.91^*^	0.45–1.36	2000–07	3.75^*^	3.36–4.15	2007–11	−1.12	−2.38–0.15	2011–14	0.97	−1.53–3.55	2014–19	−1.4^*^	−1.94 to −0.85
Singapore	−1.14^*^	−1.36 to −0.91	2000–03	−2.71^*^	−3.77 to −1.63	2003–08	−0.4	−1.05–0.26	2008–19	−1.04^*^	−1.15 to −0.93			
Slovakia	−0.08	−0.34–0.18	2000–04	−1.64	−2.19 to −1.08	2004–12	1.73	1.51–1.96	2012–15	−0.2	−1.69–1.32	2015–19	−1.99	−2.44 to −1.53
Slovenia	−0.58	−0.86 to −0.3	2000–10	−0.22	−0.43 to −0.01	2010–14	−2.3	−3.5 to −1.08	2014–19	0.09	−0.43–0.62			
South Africa	−0.32^*^	−0.48 to −0.16	2000–05	−0.82^*^	−1.12 to −0.51	2005–10	1.19^*^	0.78–1.61	2010–16	0.2	−0.07–0.47	2016–19	−2.99^*^	−3.54 to −2.44
Spain	−1.66^*^	−1.83 to −1.48	2000–07	−2.87^*^	−3 to −2.73	2007–12	−2.02^*^	−2.35 to −1.7	2012–15	−1.35^*^	−2.37 to −0.31	2015–19	0.72^*^	0.4–1.04
Sri Lanka	0.05	−0.09–0.19	2000–12	0.75^*^	0.58–0.91	2012–19	−1.12^*^	−1.43 to −0.82						
Suriname	0.36^*^	0.02–0.7	2000–02	4.01^*^	1.76–6.3	2002–11	−0.82^*^	−1.04 to −0.61	2011–14	2.23^*^	0.41–4.1	2014–19	−0.06	−0.43–0.31
Sweden	−1.53^*^	−1.64 to −1.43	2000–04	−0.86^*^	−1.24 to −0.48	2004–14	−2.37^*^	−2.48 to −2.26	2014–19	−0.39^*^	−0.65 to −0.12			
Switzerland	−1.69^*^	−1.83 to −1.55	2000–13	−2.32^*^	−2.37 to −2.28	2013–16	−0.59	−1.46–0.29	2016–19	−0.01	−0.43–0.41			
Syrian Arab Republic	−0.13	−0.48–0.22	2000–03	−3.24^*^	−4.21 to −2.26	2003–06	−0.98	−2.92–0.99	2006–10	2.19^*^	1.26–3.13	2010–19	0.19^*^	0.05–0.34
Tajikistan	0.75^*^	0.54–0.97	2000–09	0.79^*^	0.61–0.98	2009–17	1.6^*^	1.35–1.85	2017–19	−2.75^*^	−4.5 to −0.97			
Thailand	−0.94^*^	−1.16 to −0.73	2000–02	−4.09^*^	−6.04 to −2.1	2002–13	−1.22^*^	−1.36 to −1.08	2013–19	0.64^*^	0.38–0.9			
Trinidad and Tobago	−0.8^*^	−1.1 to −0.5	2000–08	−0.81^*^	−1.12 to −0.49	2008–12	−2.17^*^	−3.5 to −0.82	2012–19	0	−0.34–0.34			
Turkey	−0.72	−1.77–0.34	2000–02	13.51^*^	7.43–19.94	2002–05	−0.51	−5.14–4.33	2005–08	−6.01^*^	−10.42 to −1.37	2008–19	−1.71^*^	−2.02 to −1.41
Turkmenistan	0.29	−0.34–0.92	2000–02	−0.54	−4.05–3.11	2002–06	2.47^*^	0.77–4.21	2006–09	−2.97	−5.98–0.12	2009–19	0.58^*^	0.36–0.8
Ukraine	0.23	−0.13–0.59	2000–09	−1.71^*^	−2.31 to −1.12	2009–19	2.01^*^	1.5–2.52						
United Kingdom	−0.73^*^	−0.9 to −0.56	2000–02	−0.59	−1.86–0.69	2002–08	−1.78^*^	−2.06 to −1.5	2008–14	−0.53^*^	−0.8 to −0.25	2014–19	0.24	−0.02–0.5
United States of America	−1.22^*^	−1.38 to −1.06	2000–04	−2.71^*^	−3.01 to −2.4	2004–10	−1.99^*^	−2.21 to −1.77	2010–13	−1.05^*^	−2 to −0.1	2013–19	0.48^*^	0.33–0.63
Uruguay	−0.8^*^	−1.01 to −0.59	2000–04	1.21^*^	0.67–1.76	2004–09	−1.28^*^	−1.8 to −0.75	2009–16	−2.16^*^	−2.44 to −1.88	2016–19	0.56	−0.29–1.41
Uzbekistan	0.52^*^	0.21–0.83	2000–03	3.44^*^	1.42–5.49	2003–19	−0.02	−0.16–0.13						
Venezuela, Bolivarian Republic of	0.17	−0.6–0.96	2000–08	2.23^*^	1.13–3.34	2008–15	−3.78^*^	−5.17 to −2.38	2015–19	3.23^*^	0.77–5.74			

* *p-value < 0.05*.

*AAPC, Average Annual Percentage Change; EAPC, Estimated Annual Percentage Change*.

### Trend Patterns in Most Recent Period

**Figure 4:**
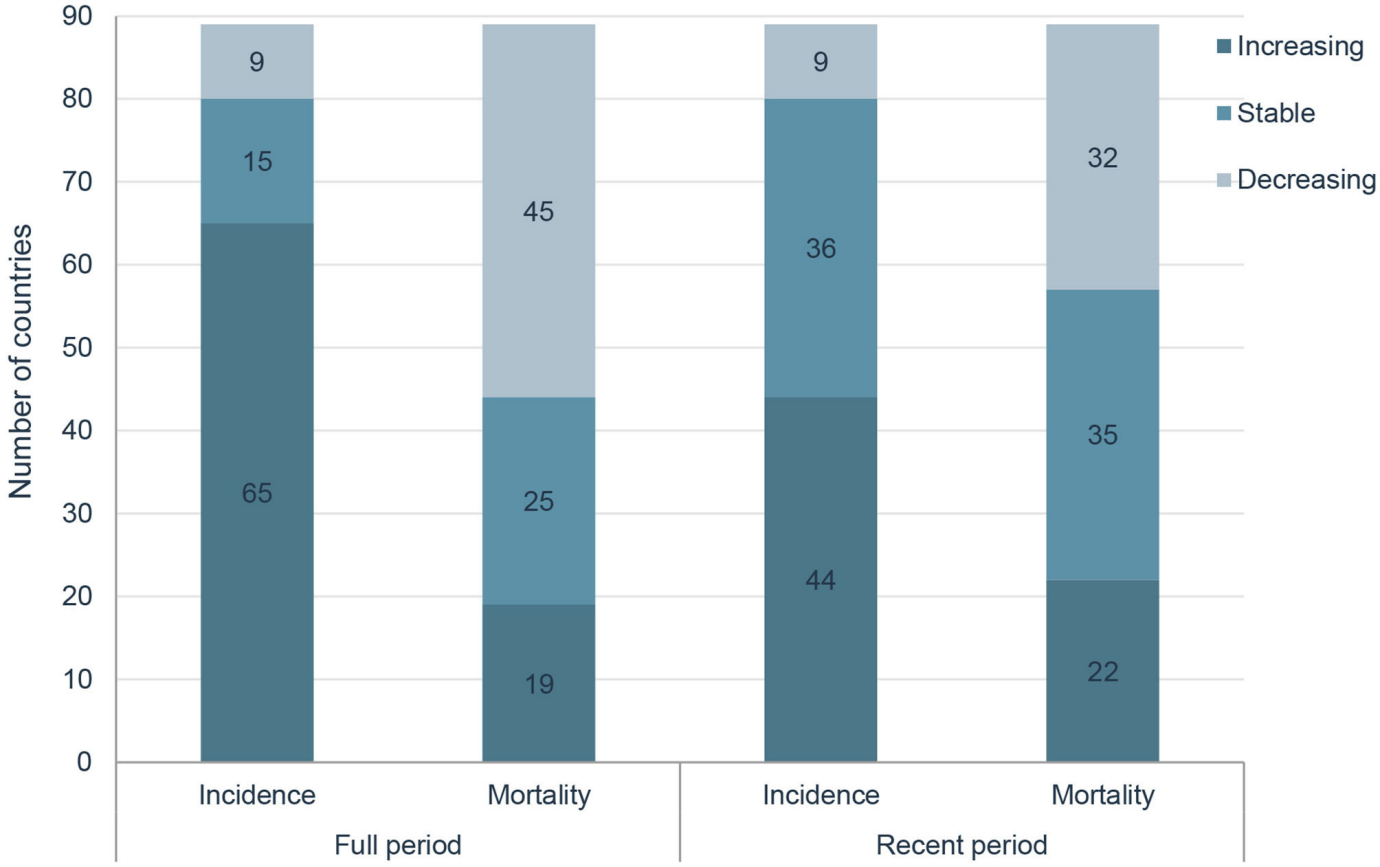
Comparisons of changing trends in most recent period with that in full period.

However, in the latest period, the magnitudes of increase for ASIRs and decrease for ASMRs were attenuated or even reversed. ASIRs have been significantly increasing in only 44 countries in the most recent period compared with 65 countries in the full period, and ASMRs have been significantly decreasing in 32 countries in the most recent period compared with 45 countries in the full period (Figure 4). For instance, the AAPC of ASIR in the United States of America was −0.69% (95% CI: −0.92 to −0.46%) from 2000 to 2019, while the AAPC was 0.49% (95% CI: 0.06–0.91%) from 2015 to 2019. And the AAPC of ASMR was −1.22% (95% CI: −1.38 to −1.06%) from 2000 to 2019 but it was 0.48% (95% CI: 0.33–0.63%) from 2015 to 2019 (Tables 3, 4).

## Discussion

In this study, we carried out a comprehensive analysis to examine the global status and temporal trends in prostate cancer incidence and mortality in 89 countries, and the results showed that HDI was positively correlated with ASIRs and negatively correlated with ASMRs. In the past decades, ASIRs have been increasing in 65 countries, stable in 15 countries and decreasing in 9 countries, and ASMRs have been increasing in 19 countries, stable in 25 countries and decreasing in 45 countries. However, in the latest period, the magnitudes of increase for ASIRs and decrease for ASMRs were attenuated or even reversed.

Globally, tremendously international variations exist in the prostate cancer incidence rates, including higher incidence rates of prostate cancer in Northern America, Europe, and Oceania, lower rates in Africa and Asia. In addition, a positive correlation between the incidence of prostate cancer and HDI, which is consistent with previous studies (2, 42, 43). The occurrence and progression of prostate cancer experienced complicated process under both genetic and environmental influences. Except for age, which was adjusted in our study, race disparity was documented in a few studies. The AISRs in several countries in Caribbean like Barbados, Saint Lucia, Bahamas and Trinidad and Tobago exceeded 89 per 100,000, which could be partly attributed to the African ancestry (29, 44, 45). Similar race disparity was also determined in USA, the incidence rate of prostate cancer in the Black men is nearly 1.7 times higher than that in White men, while the relative survival rate is lower for Black men than White men (46, 47).

Though PSA-based screening is still controversial, widely adopted PSA screening may play an important role in the increasing ASIRs in countries with very high or high HDI, like USA, UK, Canada, Australia, Swiss, Sweden, Japan. Results from three representative trials have documented the effect of PSA screening in increasing the incidence of prostate cancer, by 12% from the Prostate, Lung, Colorectal and Ovarian (PLCO) screening trial (48), 91% from the European Randomized Study of Screening for Prostate Cancer (ERSPC) (49), and 51% from the Göteborg Randomized Population-based Prostate Cancer Screening Trial (50). The effect of overdiagnosis of prostate cancer from PSA screening has been discussed in both population-based and modeling studies, ranging from 1.7 to 67% (16). However, increasing trends of ASIRs were also found in several Asia countries without national organized screening programs, like China, Thailand, Georgia and Korea. Reasons for the increase are unclear, and a few studies suggested some potential risk factors, including increasing prevalence of obesity and diabetes mellitus, westernized diet, and inadequate physical activity (51–55). Taking China for example, the prevalence of overweight and obesity has been increasing from 29.9 to 50.7% in the past two decades, and it was expected to reach 65.3% by 2030 (56).

Unlike ASIR, the ASMR decreased with the HDI, and ASMR continued to decrease or kept stable in 70 out of 89 countries, which reflects the effect of widespread PSA screening, active surveillance of high-risk population and improved treatment regimens for prostate cancer. As previous studies stated (1–3), PSA screening have been adopted since 1990's in quite a few countries in America, Europe, and Australia, which detects more prostate cancer in localized stage, resulting in the downward of advanced stage of prostate cancer, and the five-year survival rates of prostate cancer in these countries have been approaching 100% (57). Evidence from randomized trials have also demonstrated the effect of PSA screening in mortality reduction of prostate cancer, by 20% at 16 year of follow-up from ERSPS (49) and by 35% at 18 year of follow-up from Göteborg screening trial (50). In addition, for several countries in Asia and Africa, the ASMR continued to decrease, and the corresponding survival rates have been increasing in the past two decades. Taking China for example, the relative survival rate of prostate cancer rose from 53.8% in 2003–2005 to 66.4% in 2012–2015 (58), and no national prostate cancer screening program was implemented during the period. Notably, ASMRs of prostate cancer have been increasing in 19 counties, most from Asia and Central and Eastern Europe. The underlying reasons are unclear, and the increasing risk factors for aggressive prostate cancer might partly explain, such as increased intake of energy, animal fat and red meats (2, 52).

Notably, in USA, Canada, and some countries in Europe, the ASIRs are decreasing and ASMRs are increasing. It is still undefined whether the recommendations against the PSA screening from several guidelines play the crucial role. In USA, the introduction of PSA into the population in the early 1990, and then prostate cancer incidence and mortality has been decreasing (10). Because of concerns about over detection, treatment morbidity, and limited short-term absolute mortality benefit, the US Preventive Services Task Force (USPSTF) recommended against PSA screening for men aged 75 years and older in 2008 and for all ages in 2012 (25, 59), Thereafter, the screening rates of PSA have been continued to be decreasing in men aged 50 years and older (10, 26). After a decline in PSA test usage, there has been an increased burden of late-stage disease, and the decline in prostate cancer mortality has leveled off (10). Modeling studies demonstrated that newly diagnosed metastatic prostate cancer cases increased by 44%-60% in the 5 years after the 2012 USPSTF recommendation (30). Similar inversions in incidence and mortality of prostate cancer were also documented by previous studies, along with steady declines of willingness in physicians and general population toward PSA screening test after the changes in the USPSTF's recommendations in Canada and Australia (27, 60). In 2018, the USPSTF recommended discussion of the potential benefits and harms of screening with their clinician for men aged 55–69 years (31). Subsequently, a few more screening guidelines have been published in support of PSA screening (32–34). Herein, more studies are needed to determine the effect of positive recommendations of PSA screening. In addition, with the advent of personalized medicine and development of multi-omics analysis, a few genomic, epigenomic, and transcriptomic biomarkers have been identified to be potentially associated with prostate cancer risk, while the utility of these biomarkers in the screening and surveillance is unclear, and the head-to-head comparison with PSA is lacking (61–64).

### Strengths and Limitations

The main strength of our study is the effective integration of two public databases with high-quality, thus to provide the latest status and temporal changes of prostate cancer throughout the world. However, some limitations are noteworthy when interpreting the results from our study. First, the availability and quality of original data in the GBD platform were inconsistent for different countries, and some data were indirectly estimated from modeling strategy (37). Second, though we separately analyzed the current status and temporal trends by using the GLOBOCAN 2020 and GBD 2019 platform, respectively, the differences still existed for two platform in data source, analysis method and covariates for modeling epidemiologic data (65). Third, most countries included in our studies had high or very high HDI, which might apparently limit the representativeness and extrapolation of the results. Forth, we could not acquire the accurate information of prostate cancer screening program in individual country, including the population coverage, screening rates, form of implementation and health effectiveness, which may result in bias when inferring the effect of PSA screening on incidence and mortality of prostate cancer.

## Conclusions

Our study provides the up-to-date status of prostate cancer incidence and mortality worldwide, and demonstrates a positive association between prostate cancer incidence and HDI, and a negative association between prostate cancer mortality and HDI. Moreover, the magnitude of increasing incidence and decreasing mortality of prostate cancer is attenuated in the recent period. Further study is needed to analyze the absolute effects of risk factors, PSA screening and advanced treatment.

## Data Availability Statement

The original contributions presented in the study are included in the article/supplementary material, further inquiries can be directed to the corresponding authors.

## Author Contributions

LD, ZW, and LW: conception and design. LW, BL, MH, and YW: acquisition, analysis, or interpretation of data. LW, BL, and MH: drafting of the manuscript. YW, ZW, and LD: critical revision of the manuscript for important intellectual content. BL: statistical analysis. LD: administrative, technical, or material support. LD and ZW: supervision. All authors contributed to the article and approved the submitted version.

## Funding

This work was supported by Health Science and Technology Project of Zhejiang Province (2021KY586) and National Natural Science Foundation of China (12071032). The funders had no role in the study design and conduct; data collection, management, analysis, and interpretation; manuscript preparation, review, or approval; and the decision to submit the manuscript for publication.

## Conflict of Interest

The authors declare that the research was conducted in the absence of any commercial or financial relationships that could be construed as a potential conflict of interest.

## Publisher's Note

All claims expressed in this article are solely those of the authors and do not necessarily represent those of their affiliated organizations, or those of the publisher, the editors and the reviewers. Any product that may be evaluated in this article, or claim that may be made by its manufacturer, is not guaranteed or endorsed by the publisher.
